# Umbralisib antagonizes multidrug resistance in ABCB1-overexpressing cancer cells

**DOI:** 10.3389/fonc.2026.1916572

**Published:** 2026-08-13

**Authors:** Letao Bo, Bohan Zhang, Xuan-Yu Chen, Xuanming Mao, Luke Zhong, Meizi Tan, Mingyu Cui, Harsh Patel, John N. D. Wurpel, Zhe-Sheng Chen

**Affiliations:** 1 Department of Pharmaceutical Sciences, College of Pharmacy and Health Sciences, St. John’s University, New York, NY, United States; 2 Affiliated Hospital of North China University of Science and Technology, Tangshan, Hebei, China; 3 John L. Miller Great Neck North High School, Great Neck, NY, United States; 4 Holy Trinity Diocesan High School, Hicksville, NY, United States

**Keywords:** ABC transporter, ABCB1, cancer chemotherapy, multidrug resistance, umbralisib

## Abstract

**Introduction:**

ABCB1-mediated multidrug resistance (MDR) reduces the intracellular accumulation of anticancer drugs and limits chemotherapy efficacy. This study investigated whether umbralisib, a dual PI3Kδ/CK1ϵ inhibitor, could reverse ABCB1-mediated MDR.

**Methods:**

The reversal effect of umbralisib was evaluated in ABCB1-overexpressing drug-selected and transfected cell models using MTT assays. Drug accumulation and efflux assays, ATPase analysis, molecular docking, Western blotting, immunofluorescence, apoptosis analysis, and 3D spheroid assays were performed to explore the underlying mechanism.

**Results:**

Umbralisib significantly sensitized ABCB1-overexpressing cells to paclitaxel, vincristine, and doxorubicin in a concentration-dependent manner, but did not markedly reverse ABCC1-, ABCG2-, or ABCC10-mediated MDR. Mechanistically, umbralisib increased intracellular [^3^H]-paclitaxel accumulation and inhibited its efflux without altering ABCB1 expression or membrane localization. ATPase analysis and molecular docking supported a potential interaction between umbralisib and the ABCB1 drug-binding cavity. Umbralisib also enhanced paclitaxel-induced apoptosis and restored chemosensitivity in 3D tumor spheroids.

**Conclusion:**

Umbralisib reverses ABCB1-mediated MDR mainly by inhibiting ABCB1 drug efflux rather than altering transporter expression or localization, providing mechanistic insight into the modulation of ABCB1-mediated chemoresistance.

## 1 Introduction

Cancer is a major health burden, as American Cancer Society reported, around 2 million new cancer cases and ~600,000 cancer deaths are estimated to occur in the United States (1). Despite the development of cancer treatment in recent decades, chemotherapy continues to serve as a principal treatment modality for cancer (2). The ineffectiveness of cancer chemotherapy, however, may at times arise from multidrug resistance (MDR) with the mechanism of diminished uptake and/or enhanced efflux of chemotherapeutic agents, regulation of apoptosis, drug metabolism changes, enhancement of DNA damage repair, etc. (3). Notably, the ABC transporters, located in cancer cell membranes, serve as a major mechanism of MDR associated with drug efflux (4, 5).

The ABC transporter superfamily, a major obstacle to cancer therapies, comprises various active membrane transporters that share common structural features, including nucleotide-binding domains (NBDs) and transmembrane domains (TMDs) (6). This ABC transporter family is classified into seven groups, namely ABCA through ABCG, according to domain homology as well as gene structure (7). ABCB1, also referred to as P-glycoprotein (P-gp) or multidrug resistance protein 1 (MDR1), is the earliest identified member of the ABCB family and plays a pivotal role in mediating MDR (8). ABCB1 is capable of transporting a broad spectrum of chemotherapeutic agents, including paclitaxel, vincristine, and doxorubicin, thereby lowering their intracellular concentrations and reducing cytotoxicity (9). Although direct inhibition of ABCB1 has been extensively investigated as an approach to reverse MDR, the clinical application of direct ABCB1 inhibitors has been limited by low efficacy, adverse pharmacokinetic interactions, and toxicity (10, 11). Phosphoinositide 3-kinases (PI3Ks) regulate multiple cellular processes, including proliferation, survival, metabolism, and motility, and dysregulation of PI3K signaling is closely associated with tumor progression and therapeutic resistance (12, 13). Casein kinase 1 (CK1) family members are also involved in cell cycle regulation, DNA damage response, and signal transduction, and their dysregulation has been linked to cancer development and treatment resistance (14). Therefore, dual inhibitors targeting PI3K- and CK1-related signaling may have additional pharmacological effects beyond their kinase-target activity.

Umbralisib is a dual inhibitor of PI3Kδ and CK1ϵ (15). Although it was previously developed for the treatment of hematologic malignancies, umbralisib was later withdrawn due to safety concerns (16). Nevertheless, its dual kinase-target profile prompted us to investigate whether it may also modulate ABCB1-mediated MDR. In the present study, we examined whether umbralisib could resensitize ABCB1-overexpressing cancer cells to conventional chemotherapeutic agents and explored the underlying mechanism through cytotoxicity reversal assays, drug accumulation and efflux studies, ATPase analysis, protein expression and localization assays, apoptosis evaluation, 3D spheroid experiments, and molecular docking analysis.

## 2 Materials and methods

### 2.1 Chemicals and reagents

Umbralisib (TGR-1202) was purchased from MedChemExpress (Monmouth Junction, NJ, USA) prepared as a stock solution in dimethyl sulfoxide (DMSO) and stored at −20 °C. Working solutions were freshly prepared by diluting the stock solution in complete medium immediately before use. In all treatment groups, including the corresponding controls, the final DMSO concentration was kept constant at 0.1% (v/v) or less. Paclitaxel, vincristine, doxorubicin, cisplatin, colchicine, topotecan, mitoxantrone, verapamil, MK571, Ko143, cepharanthine, and G418 were all acquired from Sigma-Aldrich (St. Louis, MO, USA). [^3^H]-paclitaxel was purchased from Moravek Biochemicals (Brea, CA, USA). MTT was purchased from Fisher Scientific (NJ, USA). DMSO, Triton X-100, paraformaldehyde, and other routine biochemical reagents were purchased from standard commercial sources. Antibodies against ABCB1 and GAPDH, HRP-conjugated and fluorescent secondary antibodies, and DAPI were obtained from the same vendors used in the corresponding laboratory procedures. All reagents used in this work were of analytical grade or above.

### 2.2 Cell lines and cell culture

A panel of parental and drug-resistant cell lines was used to evaluate the reversal effect on ABC transporter-mediated multidrug resistance. KB-3-1 (parental) and KB-C2 cells (ABCB1 overexpression, colchicine-selected); SW620 (parental) and SW620/Ad300 cells (ABCB1 overexpression, doxorubicin-selected); HEK293 cells transfected with ABCB1 and empty vector control. ABCC1-overexpressing KB-CV60 cells; ABCG2-overexpressing H460/MX20 cells; HEK293 cells transfected with ABCC10 and vector control. Resistant cells were cultured in medium with selective agents (e.g., colchicine, doxorubicin, vincristine, topotecan, mitoxantrone). Prior to experiments, the drug-selected cells grow in the absence of selective drugs for at least 2 weeks, and transfected cells were cultured without G418 for 1 week. All cells were grown as adherent monolayers in DMEM (10% FBS, 37°C, 5% CO_2_) with antibiotics and passaged using trypsin-EDTA during logarithmic growth.

### 2.3 Cytotoxicity assay

An MTT colorimetric assay was used to evaluate the reversal of ABC transporter-mediated multidrug resistance. Cells were seeded in 96-well plates, pretreated with test compounds (e.g., umbralisib) or reference inhibitors (verapamil, MK571, Ko143, cepharanthine), and then exposed to serial dilutions of chemotherapeutic agents (paclitaxel, vincristine, doxorubicin, or cisplatin as control). The final DMSO concentration was kept constant at 0.1% (v/v) or less across all treatment and control groups. After 68 h, MTT was added. 4 h later, formazan crystals were dissolved in DMSO, and absorbance was measured at 570 nm. IC_50_ values and resistance fold were calculated from dose-response curves. All experiments were independently repeated three times.

### 2.4 [^3^H]-Paclitaxel accumulation and efflux assay

The [^3^H]-paclitaxel accumulation and efflux assays were performed to determine whether umbralisib modulates ABCB1-mediated efflux function in KB-3–1 and KB-C2 cells. KB-3–1 and KB-C2 cells were seeded in 24-well plates and preincubated with umbralisib (3 or 10 μmol/L) or verapamil for 2 h. For the accumulation assay, 1 nmol/L [³H]-paclitaxel was added, and the cells were incubated at 37 °C for an additional 2 h. After washing with ice-cold PBS, the cells were collected, and intracellular radioactivity was measured using a Packard TRI-CARB 1900CA liquid scintillation analyzer. For the efflux assay, [^3^H]-paclitaxel-loaded cells were washed and incubated in fresh medium containing the corresponding treatment. Residual intracellular radioactivity was determined at 0, 30, 60, and 120 min and expressed as a percentage of the initial value. All experiments were independently repeated three times.

### 2.5 ATPase assay

To measure ABCB1 ATPase activity, we prepared crude membranes enriched for the transporter according to our standard laboratory procedure. The membrane vesicles were subsequently exposed to different concentrations of umbralisib in ATPase assay buffer. Sodium orthovanadate was used to define the vanadate-sensitive component of the activity. Basal ATPase activity was defined as the activity detected in the absence of umbralisib and was normalized to 100%. ATPase values obtained under other conditions were expressed as percentages of this control. The net activity was calculated as total ATPase activity minus the vanadate-insensitive fraction. All experiments were independently repeated three times.

### 2.6 Western blot analysis

Western blot analysis was performed to determine whether umbralisib affects ABCB1 protein expression in resistant cancer cells. Cells were treated with umbralisib at 1–10 μmol/L for up to 72 h, and ABCB1 protein levels were analyzed by immunoblotting. Cells were lysed in RIPA buffer containing protease inhibitors, and total protein concentrations were determined using the Bradford assay. Equal amounts of protein (30 μg per lane) were separated by 10% SDS-PAGE and transferred onto PVDF membranes. After blocking with 5% non-fat milk in TBST for 1 h, the membranes were incubated overnight at 4 °C with primary antibodies against ABCB1 and GAPDH, followed by HRP-conjugated secondary antibody incubation for 1 h at room temperature. Protein bands were detected using an enhanced chemiluminescence system, and band intensities were quantified with ImageJ software. ABCB1 expression was normalized to the corresponding GAPDH signal. All experiments were independently repeated three times.

### 2.7 Immunofluorescence analysis

Immunofluorescence analysis was performed to examine whether umbralisib affects the subcellular localization of ABCB1. Cells were incubated with umbralisib at 10 μmol/L for up to 72 h. Cells were seeded on sterile coverslips in 24-well plates and allowed to attach for 24 h. After treatment with umbralisib, the cells were fixed with 4% paraformaldehyde for 15 min, permeabilized with 0.1% Triton X-100 for 10 min, and blocked with 5% bovine serum albumin for 1 h at room temperature. The cells were then incubated overnight at 4 °C with an anti-ABCB1 primary antibody, followed by incubation with a fluorescent secondary antibody for 1 h in the dark. Nuclei were counterstained with DAPI, and fluorescence images were acquired using identical imaging settings. ABCB1-associated fluorescence was analyzed using ImageJ software. All experiments were independently repeated three times.

### 2.8 Flow cytometric analysis of apoptosis

Annexin V/PI staining followed by flow cytometry was used to evaluate apoptosis in KB-3–1 and KB-C2 cells. After overnight attachment in 6-well plates, cells were treated with umbralisib, paclitaxel, or both agents together. The final DMSO concentration was kept constant at 0.1% (v/v) or less across all treatment and control groups. At the end of treatment, all cells, including those in suspension and those attached to the culture plate, were harvested, washed twice with ice-cold PBS, and resuspended in binding buffer. Cells were then stained with Annexin V and PI in accordance with the manufacturer’s instructions, and the percentages of viable, early apoptotic, late apoptotic, and necrotic populations were determined using flow cytometry software. Cellular debris was excluded based on forward- and side-scatter characteristics, followed by singlet gating using FSC-A and FSC-H. Annexin V/PI quadrant gates were established using unstained and single-stained controls. All experiments were independently repeated three times.

### 2.9 Three-dimensional multicellular spheroid assay

To evaluate the reversal effect of umbralisib in a three-dimensional model, multicellular spheroids were generated from SW620 and SW620/Ad300 cells using a non-adherent culture method. U-bottom 96-well plates were coated with agarose to prevent cell attachment, and 300 cells/well were seeded for spheroid formation. After uniform spheroids formed, the treatment groups were initiated with vehicle, umbralisib, paclitaxel, paclitaxel plus umbralisib, or paclitaxel plus verapamil. The final DMSO concentration was kept constant at 0.1% (v/v) or less across all treatment and control groups. Spheroid morphology and growth were monitored by bright-field microscopy, and images were captured up to 6 days using the same magnification. Spheroid images were analyzed using ImageJ software. The longest diameter (D_max_) and the perpendicular shortest diameter (D_min_) of each spheroid were measured using ImageJ. Assuming an ellipsoidal shape with equal width and depth, spheroid volume was estimated using the equation V=(π/6)×D_max_×D_min_^2^. The percentage change in spheroid volume relative to baseline was determined using the following equation: [(V_t_ – V_0_)/V_0_] × 100%, where V_t_ represents the spheroid volume at the indicated time point and V_0_ represents the initial spheroid volume. All experiments were independently repeated three times.

### 2.10 Docking analysis

Molecular docking was performed to evaluate the interaction between umbralisib and the ABCB1 drug-binding pocket using the same workflow established in our laboratory. The ABCB1 model was processed using Maestro’s Protein Preparation Wizard module. (Schrödinger, LLC, Cambridge, MA, USA). The docking grid was generated at the transmembrane drug-binding cavity of ABCB1. The chemical structure of umbralisib was prepared using the LigPrep module with default settings. Glide XP docking was then carried out using the processed ligand and protein model. The top-ranked binding poses were analyzed for potential binding interactions within the substrate-binding pocket.

### 2.11 Statistical analysis

Data are expressed as mean ± SD. Statistical analysis was conducted using GraphPad Prism. Differences between two groups were evaluated with Student’s t-test, while comparisons involving more than two groups were analyzed by one-way ANOVA followed by an appropriate *post hoc* test. A P value of less than 0.05 was considered statistically significant.

## 3 Results

### 3.1 Umbralisib reversed ABCB1-mediated MDR in ABCB1-overexpressing cancer cells

**Figure 1:**
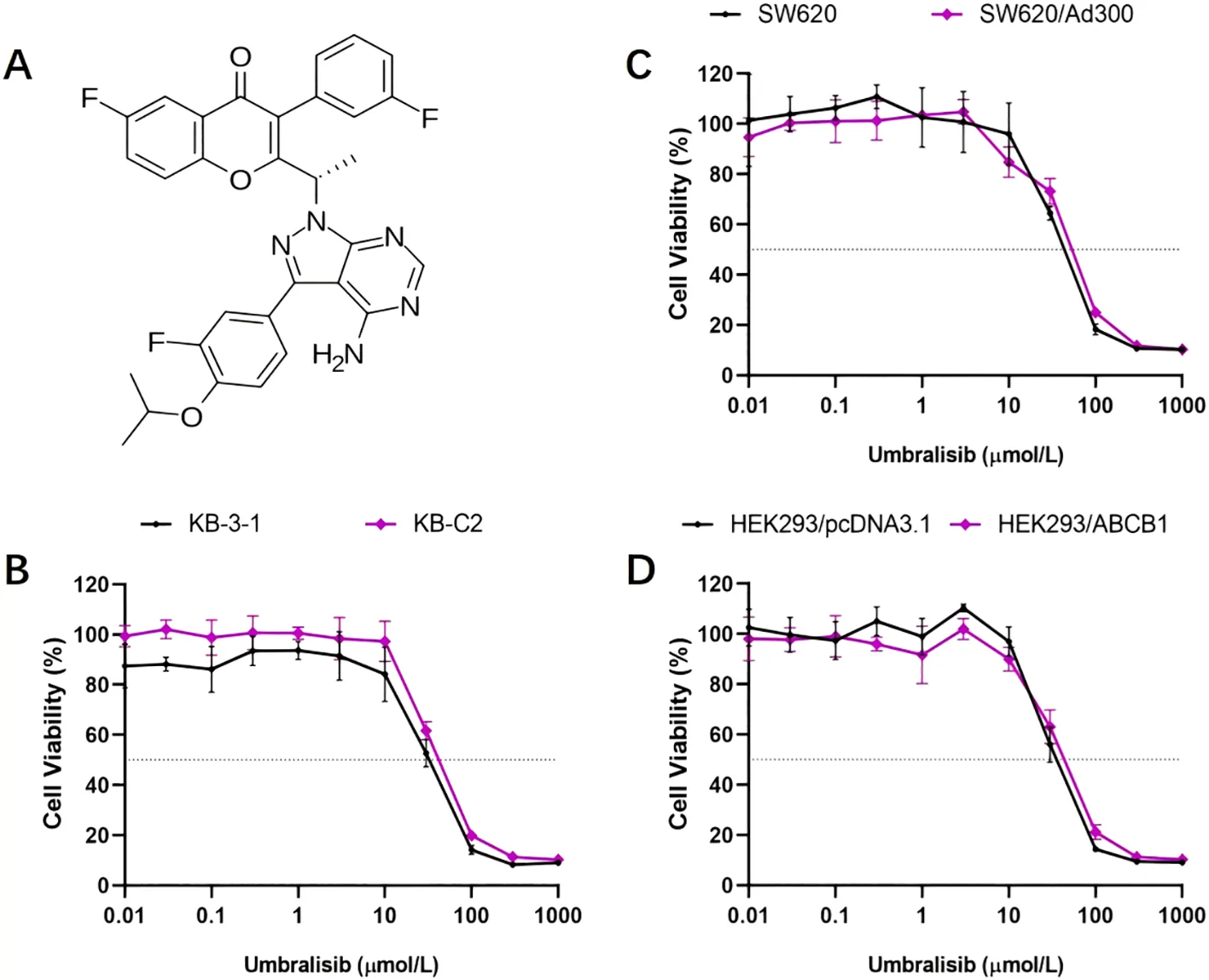
Impact of umbralisib on viability in parental and ABCB1-overexpressing cell models. Cell survival was determined using the MTT assay following 72 h exposure to escalating concentrations of umbralisib. **(A)** Chemical structure of umbralisib. **(B)** The parental KB-3–1 line and its ABCB1-overexpressing resistant counterpart, KB-C2. **(C)** The parental SW620 line and its ABCB1-overexpressing resistant counterpart, SW620/Ad300. **(D)** Vector-control HEK293/pcDNA3.1 cells and ABCB1-transfected HEK293/ABCB1 cells. Results are expressed as mean ± SD with three independent experiments.

First, to identify non-toxic concentrations of umbralisib, we evaluated its cytotoxicity in the cell lines used in this study. Concentrations below the IC_20_ after 72 h of incubation were selected to minimize any significant effect on cell viability. Based on these results (**Figure 1**), the subsequent assays were performed using umbralisib at 1, 3, and 10 μmol/L.

**Figure 2:**
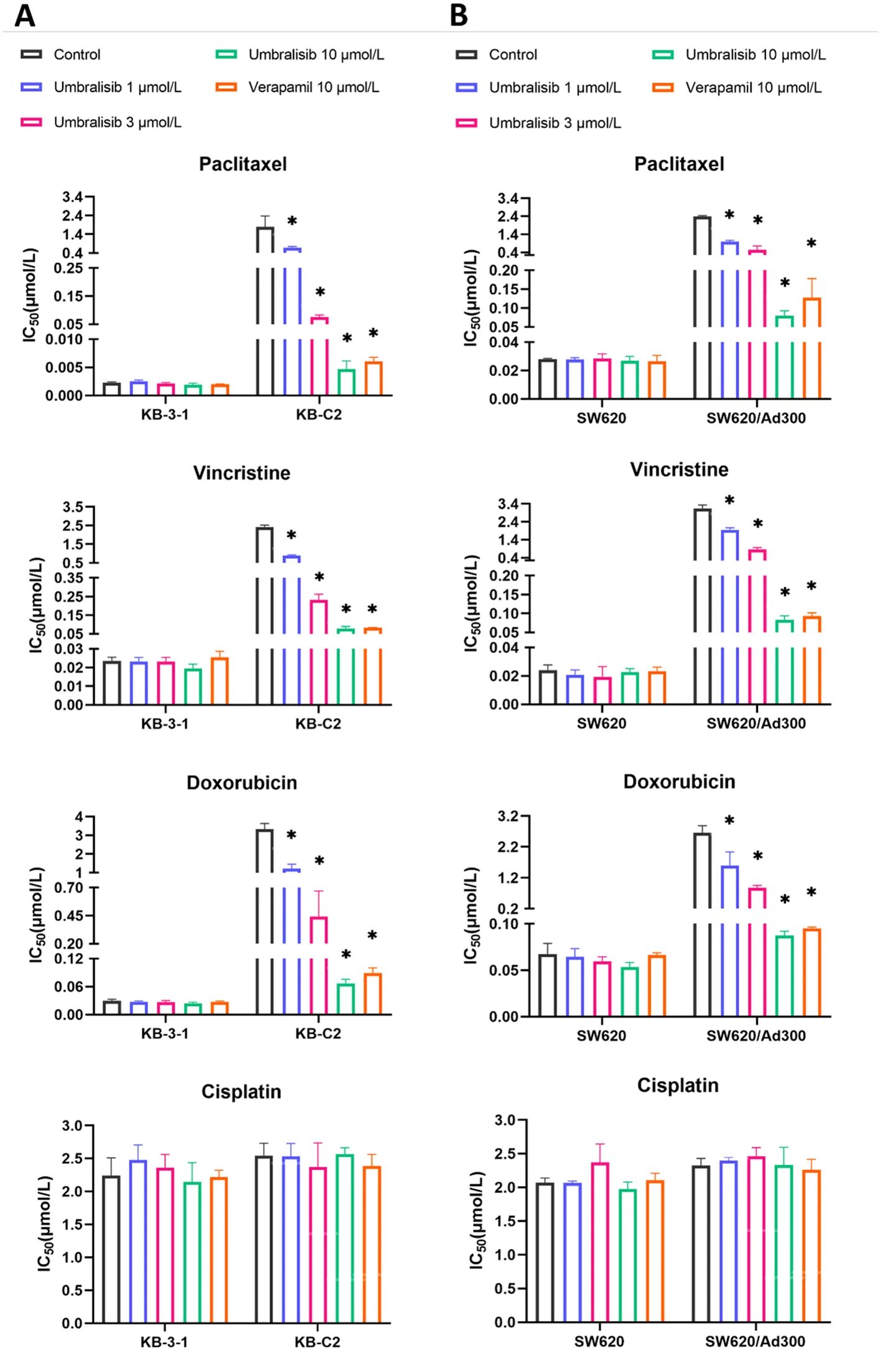
Umbralisib enhanced chemosensitivity of ABCB1-overexpressing cells to transporter-substrate drugs. **(A)** IC_50_ values of paclitaxel, doxorubicin, vincristine, and cisplatin in parental KB-3–1 cells and ABCB1-overexpressing KB-C2 cells treated with each chemotherapeutic drug with or without umbralisib (1, 3, or 10 μmol/L) for 72 h. **(B)** IC_50_ values of ABCB1-substrate drugs (paclitaxel, doxorubicin, and vincristine) and cisplatin in parental SW620 cells and ABCB1-overexpressing SW620/Ad300 cells treated in the absence or presence of umbralisib (1, 3, or 10 μmol/L) for 72 h. Verapamil (10 μmol/L) served as the positive reversal control. Results are expressed as mean ± SD with three independent experiments. *P < 0.05 compared with the control group.

To determine whether umbralisib could reverse ABCB1-mediated MDR in drug-selected cancer cell models, parental KB-3–1 and SW620 cells and their corresponding ABCB1-overexpressing KB-C2 and SW620/Ad300 cells were treated with paclitaxel, vincristine, doxorubicin, or cisplatin when treated with or without umbralisib. As shown in **Figure 2**, umbralisib significantly sensitized KB-C2 and SW620/Ad300 cells to paclitaxel, vincristine, and doxorubicin in a concentration-dependent manner while producing no significant effect in parental cells. In KB-C2 cells, the resistance fold of paclitaxel decreased from 782.91 to 295.87, 32.83, and 2.04 in the presence of 1, 3, and 10 μmol/L umbralisib, respectively (**Table 1**). In SW620/Ad300 cells, the resistance fold of paclitaxel decreased from 85.11 to 35.18, 19.79, and 2.86 under the same concentration-dependent treatment. Similar concentration-dependent sensitization was observed for vincristine and doxorubicin in both resistant cell lines.

**Table 1:** Umbralisib reverses ABCB1-mediated MDR in drug-resistant cancer cells.

Treatment	IC_50_ value ± SD^a^ (μmol/L, Resistance fold^b^)			
	KB-3-1 (Parental)	KB-C2 (Resistant)	SW620 (Parental)	SW620/Ad300 (Resistant)
Paclitaxel	0.0023 ± 0.0001 (1.00)	1.8007 ± 0.5729 (782.91)	0.028 ± 0.001 (1.00)	2.383 ± 0.060 (85.11)
+Umbralisib 1 μmol/L	0.0025 ± 0.0002 (1.09)	0.6805 ± 0.0674 (295.87)	0.028 ± 0.001 (1.00)	0.985 ± 0.085 (35.18)
+Umbralisib 3 μmol/L	0.0021 ± 0.0002 (0.91)	0.0755 ± 0.0077 (32.83)	0.029 ± 0.003 (1.04)	0.554 ± 0.201 (19.79)
+Umbralisib 10 μmol/L	0.0019 ± 0.0002 (0.83)	0.0047 ± 0.0014 (2.04)	0.027 ± 0.003 (0.96)	0.080 ± 0.013 (2.86)
+Verapamil 10 μmol/L	0.0020 ± 0.0001 (0.87)	0.0060 ± 0.0007 (2.61)	0.027 ± 0.004 (0.96)	0.128 ± 0.050 (4.57)
Vincristine	0.024 ± 0.002 (1.00)	2.410 ± 0.114 (100.42)	0.024 ± 0.004 (1.00)	3.134 ± 0.194 (130.58)
+Umbralisib 1 μmol/L	0.023 ± 0.002 (0.96)	0.888 ± 0.043 (37.00)	0.021 ± 0.004 (0.88)	1.953 ± 0.119 (81.38)
+Umbralisib 3 μmol/L	0.023 ± 0.002 (0.96)	0.232 ± 0.030 (9.67)	0.019 ± 0.007 (0.79)	0.857 ± 0.111 (35.71)
+Umbralisib 10 μmol/L	0.019 ± 0.002 (0.79)	0.079 ± 0.011 (3.29)	0.023 ± 0.002 (0.96)	0.084 ± 0.010 (3.50)
+Verapamil 10 μmol/L	0.025 ± 0.003 (1.04)	0.083 ± 0.002 (3.46)	0.023 ± 0.003 (0.96)	0.093 ± 0.009 (3.88)
Doxorubicin	0.030 ± 0.003 (1.00)	3.337 ± 0.300 (111.23)	0.067 ± 0.012 (1.00)	2.649 ± 0.237 (39.54)
+Umbralisib 1 μmol/L	0.028 ± 0.002 (0.93)	1.211 ± 0.244 (40.37)	0.065 ± 0.009 (0.97)	1.584 ± 0.451 (23.64)
+Umbralisib 3 μmol/L	0.027 ± 0.004 (0.90)	0.442 ± 0.228 (14.07)	0.060 ± 0.005 (0.90)	0.879 ± 0.077 (13.12)
+Umbralisib 10 μmol/L	0.024 ± 0.003 (0.80)	0.067 ± 0.009 (2.23)	0.054 ± 0.005 (0.81)	0.088 ± 0.004 (1.31)
+Verapamil 10 μmol/L	0.027 ± 0.002 (0.90)	0.089 ± 0.011 (2.97)	0.066 ± 0.002 (0.99)	0.095 ± 0.001 (1.42)
Cisplatin	2.239 ± 0.269 (1.00)	2.541 ± 0.191 (1.13)	2.072 ± 0.067 (1.00)	2.326 ± 0.102 (1.12)
+Umbralisib 1 μmol/L	2.475 ± 0.232 (1.11)	2.532 ± 0.196 (1.13)	2.064 ± 0.029 (1.00)	2.396 ± 0.047 (1.16)
+Umbralisib 3 μmol/L	2.362 ± 0.199 (1.05)	2.372 ± 0.363 (1.06)	2.368 ± 0.276 (1.14)	2.458 ± 0.132 (1.19)
+Umbralisib 10 μmol/L	2.146 ± 0.290 (0.96)	2.566 ± 0.096 (1.15)	1.975 ± 0.104 (0.95)	2.330 ± 0.263 (1.12)
+Verapamil 10 μmol/L	2.215 ± 0.106 (0.99)	2.384 ± 0.177 (1.06)	2.104 ± 0.105 (1.02)	2.258 ± 0.159 (1.09)

Resistance fold was calculated by dividing the IC_50_ values of substrates in the presence or absence of inhibitor by the IC_50_ of parental cells without inhibitor.

Data are presented as mean ± SD from three independent biological experiments.

**Figure 3:**
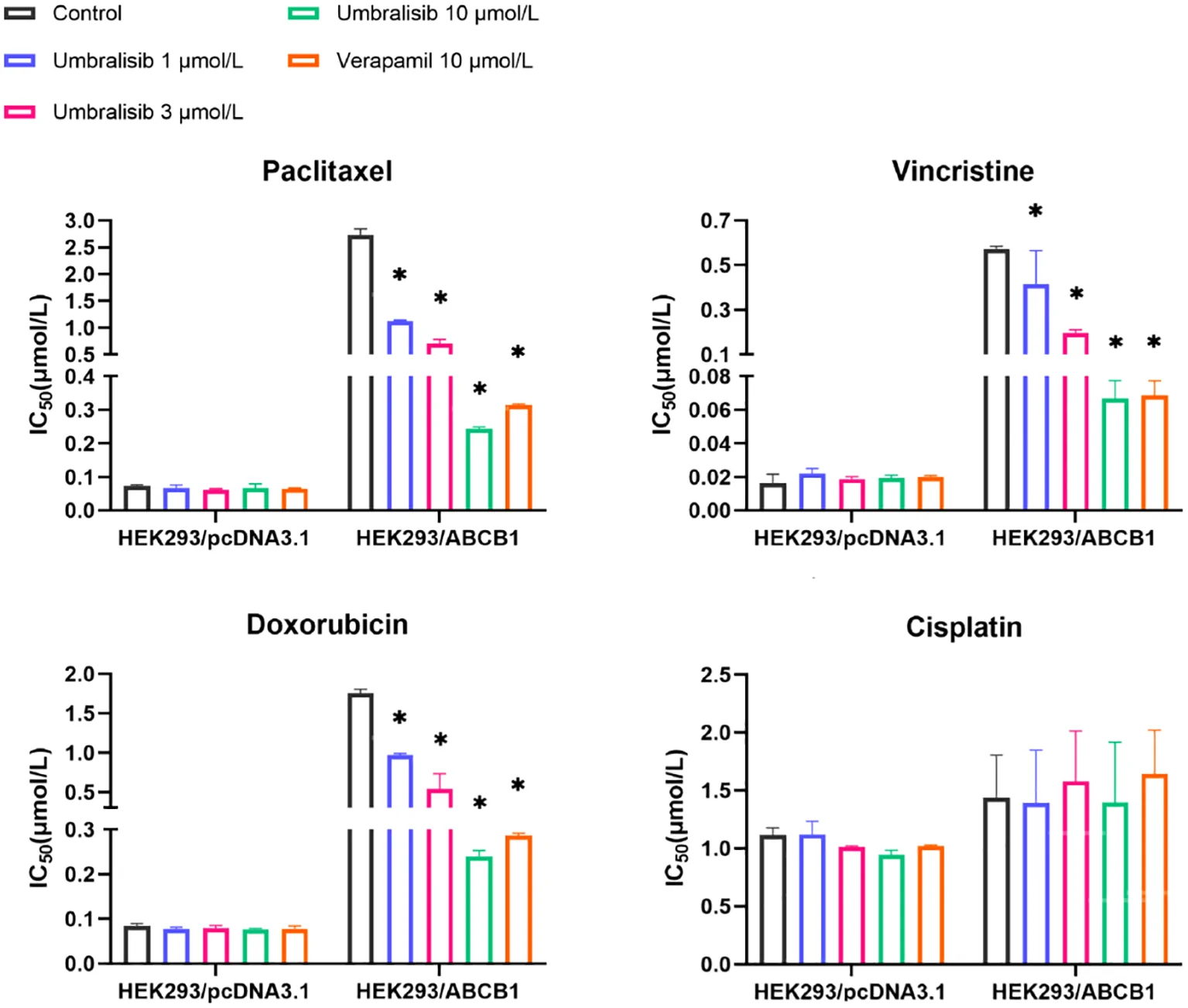
Reversal effect of umbralisib in ABCB1-transfected HEK293 cells. IC50 values for paclitaxel, doxorubicin, vincristine, and cisplatin were obtained by MTT assay after 72 h exposure of HEK293/pcDNA3.1 and HEK293/ABCB1 cells to each chemotherapeutic agent with or without umbralisib (1, 3, or 10 μmol/L). Verapamil (10 μmol/L) was included as the positive control. Values are presented as mean ± SD with three independent experiments. *P < 0.05 compared with the corresponding untreated control group.

Next, we verify the reversal effect of umbralisib in an ABCB1 gene-transfected cell model. As shown in **Figure 3**, Co-incubation with umbralisib significantly sensitized HEK293/ABCB1 cells to paclitaxel, vincristine, and doxorubicin in a concentration-dependent manner, with no significant change in the corresponding HEK293/pcDNA3.1 cells. In HEK293/ABCB1 cells, the resistance fold of paclitaxel decreased from 37.51 to 15.47, 9.63, and 3.34 in the presence of 1, 3, and 10 μmol/L umbralisib, respectively (**Table 2**). Similar concentration-dependent sensitization was observed for vincristine and doxorubicin in HEK293/ABCB1 cells.

**Table 2:** Umbralisib reverses ABCB1-mediated MDR in gene-transfected HEK293 Cells.

Treatment	IC_50_ value ± SD^a^ (μmol/L, Resistance fold^b^)	
	HEK293/pcDNA3.1 (Parental)	HEK293/ABCB1 (Resistant)
Paclitaxel	0.073 ± 0.004 (1.00)	2.738 ± 0.101 (37.51)
+Umbralisib 1 μmol/L	0.067 ± 0.009 (0.92)	1.129 ± 0.012 (15.47)
+Umbralisib 3 μmol/L	0.062 ± 0.003 (0.85)	0.703 ± 0.080 (9.63)
+Umbralisib 10 μmol/L	0.066 ± 0.013 (0.90)	0.244 ± 0.005 (3.34)
+Verapamil 10 μmol/L	0.064 ± 0.003 (0.88)	0.314 ± 0.004 (4.30)
Vincristine	0.016 ± 0.005 (1.00)	0.574 ± 0.011 (35.88)
+Umbralisib 1 μmol/L	0.022 ± 0.003 (1.38)	0.415 ± 0.151 (25.94)
+Umbralisib 3 μmol/L	0.019 ± 0.001 (1.19)	0.197 ± 0.014 (12.31)
+Umbralisib 10 μmol/L	0.019 ± 0.002 (1.19)	0.067 ± 0.010 (4.19)
+Verapamil 10 μmol/L	0.020 ± 0.001 (1.25)	0.069 ± 0.009 (4.31)
Doxorubicin	0.084 ± 0.005 (1.00)	1.760 ± 0.046 (20.95)
+Umbralisib 1 μmol/L	0.076 ± 0.004 (0.90)	0.968 ± 0.023 (11.52)
+Umbralisib 3 μmol/L	0.080 ± 0.005 (0.95)	0.542 ± 0.191 (6.45)
+Umbralisib 10 μmol/L	0.077 ± 0.002 (0.92)	0.241 ± 0.013 (2.87)
+Verapamil 10 μmol/L	0.078 ± 0.006 (0.93)	0.287 ± 0.005 (3.42)
Cisplatin	1.117 ± 0.059 (1.00)	1.437 ± 0.366 (1.29)
+Umbralisib 1 μmol/L	1.119 ± 0.115 (1.00)	1.390 ± 0.457 (1.24)
+Umbralisib 3 μmol/L	1.014 ± 0.009 (0.91)	1.578 ± 0.433 (1.41)
+Umbralisib 10 μmol/L	0.948 ± 0.037 (0.85)	1.398 ± 0.519 (1.25)
+Verapamil 10 μmol/L	1.022 ± 0.010 (0.91)	1.640 ± 0.380 (1.47)

Resistance fold was calculated by dividing the IC_50_ values of substrates in the presence or absence of inhibitor by the IC_50_ of HEK293/pcDNA3.1 cells without inhibitor.

Data are presented as mean ± SD from three independent biological experiments.

Moreover, the reversal effect of umbralisib was comparable to that of verapamil at 10 μmol/L. By contrast, umbralisib did not significantly change the IC_50_ of cisplatin (not a substrate of ABCB1) in either parental or resistant cells. These results indicate that umbralisib could reverse ABCB1-mediated MDR in ABCB1-overexpressing cancer cells in a concentration-dependent manner.

### 3.2 Umbralisib did not significantly reverse ABCC1-, ABCG2-, or ABCC10-mediated MDR

**Figure 4:**
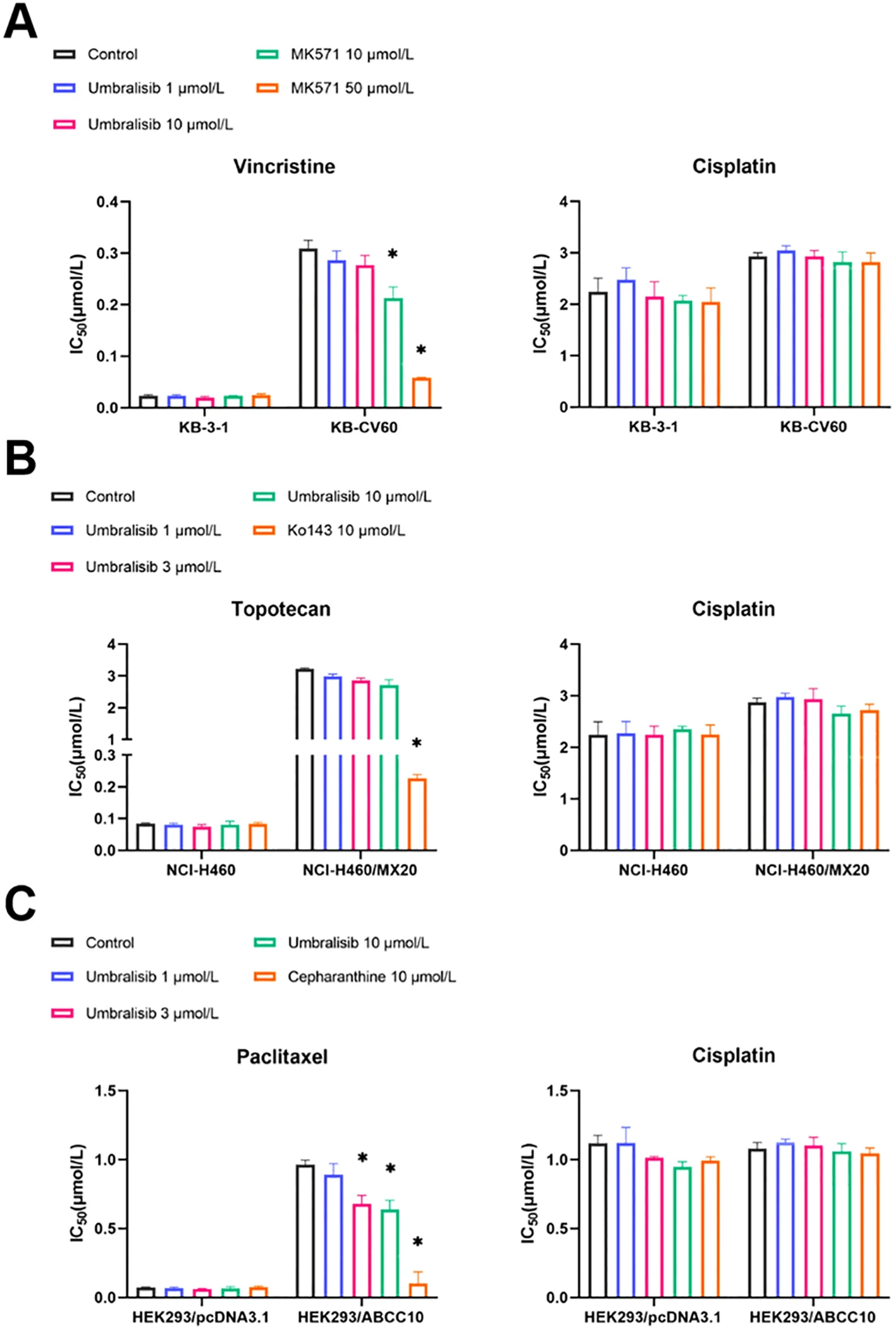
Umbralisib showed no significant reversal activity against ABCC1-, ABCG2-, or ABCC10-associated MDR. **(A)** IC_50_ values for vincristine and cisplatin were evaluated after 72 h treatment in parental KB-3–1 cells and ABCC1-overexpressing KB-CV60 cells with or without umbralisib (1 or 10 μmol/L). MK571 (10 or 50 μmol/L) served as the positive control inhibitor. **(B)** IC_50_ values for topotecan and cisplatin were measured in parental NCI-H460 cells and ABCG2-overexpressing NCI-H460/MX20 cells after 72 h exposure to umbralisib (1, 3, or 10 μmol/L). Ko143 (10 μmol/L) served as the positive control. **(C)** IC_50_ values for paclitaxel and cisplatin were determined in HEK293/pcDNA3.1 and HEK293/ABCC10 cells after 72 h treatment with umbralisib (1, 3, or 10 μmol/L). Cepharanthine (10 μmol/L) was included as the positive control. Results are expressed as mean ± SD with three independent experiments. *P < 0.05 versus the control group.

To determine whether the reversal effect of umbralisib was selective for ABCB1, we further examined its efficacy in cell models overexpressing ABCC1, ABCG2, or ABCC10. As shown in **Figure 4**, umbralisib did not significantly reverse MDR mediated by ABCC1, ABCG2, or ABCC10 under the tested conditions. In KB-CV60, NCI-H460/MX20, and HEK293/ABCC10 cells, umbralisib did not reduce the IC_50_ values of vincristine, topotecan, or paclitaxel, respectively, whereas the corresponding positive controls MK571, Ko143, and cepharanthine significantly sensitized the resistant cells (**Tables 3**–**5**). In contrast, the IC_50_ of cisplatin remained unchanged across all groups. These results suggest umbralisib effectively reversed MDR in ABCB1-overexpressing cells, while no significant reversal was observed in the ABCC1-, ABCG2-, or ABCC10-overexpressing models tested.

**Table 3:** Umbralisib did not reverse ABCC1-mediated resistance in KB-CV60 cells.

Treatment	IC _50_ value ± SD ^a^ (μmol/L, Resistance fold ^b^ )	
	KB-3-1 (Parental)	KB-CV60 (Resistant)
Vincristine	0.024 ± 0.002 (1.00)	0.309 ± 0.016 (12.88)
+Umbralisib 1 μmol/L	0.023 ± 0.002 (0.96)	0.286 ± 0.018 (11.92)
+Umbralisib 10 μmol/L	0.019 ± 0.002 (0.79)	0.277 ± 0.019 (11.54)
+MK571 10 μmol/L	0.023 ± 0.001 (0.96)	0.213 ± 0.021 (8.88)
+MK571 50 μmol/L	0.025 ± 0.002 (1.04)	0.058 ± 0.001 (2.42)
Cisplatin	2.239 ± 0.269 (1.00)	2.925 ± 0.079 (1.31)
+Umbralisib 1 μmol/L	2.475 ± 0.232 (1.11)	3.049 ± 0.089 (1.36)
+Umbralisib 10 μmol/L	2.146 ± 0.290 (0.96)	2.928 ± 0.118 (1.31)
+MK571 10 μmol/L	2.069 ± 0.099 (0.92)	2.820 ± 0.195 (1.26)
+MK571 50 μmol/L	2.047 ± 0.267 (0.91)	2.818 ± 0.180 (1.26)

Resistance fold was calculated by dividing the IC_50_ values of substrates in the presence or absence of inhibitor by the IC_50_ of KB-3–1 cells without inhibitor.

Data are presented as mean ± SD from three independent biological experiments.

**Table 4:** Umbralisib did not reverse ABCG2-mediated resistance in NCI-H460/MX20 cells.

Treatment	IC_50_ value ± SD^a^ (μmol/L, Resistance fold^b^)	
	NCI-H460 (Parental)	NCI-H460/MX20 (Resistant)
Topotecan	0.085 ± 0.002 (1.00)	3.222 ± 0.035 (37.91)
+Umbralisib 1 μmol/L	0.082 ± 0.004 (0.96)	2.980 ± 0.076 (35.06)
+Umbralisib 3 μmol/L	0.075 ± 0.006 (0.88)	2.851 ± 0.086 (33.54)
+Umbralisib 10 μmol/L	0.081 ± 0.010 (0.95)	2.717 ± 0.159 (31.96)
+Ko143 10 μmol/L	0.084 ± 0.004 (0.99)	0.227 ± 0.012 (2.67)
Cisplatin	2.240 ± 0.253 (1.00)	2.873 ± 0.082 (1.28)
+Umbralisib 1 μmol/L	2.276 ± 0.225 (1.02)	2.976 ± 0.072 (1.33)
+Umbralisib 3 μmol/L	2.241 ± 0.171 (1.00)	2.932 ± 0.208 (1.31)
+Umbralisib 10 μmol/L	2.351 ± 0.058 (1.05)	2.651 ± 0.152 (1.18)
+Ko143 10 μmol/L	2.247 ± 0.187 (1.00)	2.722 ± 0.111 (1.22)

Resistance fold was calculated by dividing the IC_50_ values of substrates in the presence or absence of inhibitor by the IC_50_ of NCI-H460 cells without inhibitor.

Data are presented as mean ± SD from three independent biological experiments.

**Table 5:** Umbralisib did not reverse ABCC10-mediated MDR in gene-transfected HEK293 cells.

Treatment	IC _50_ value ± SD ^a^ (μmol/L, Resistance fold ^b^ )	
	HEK293/pcDNA3.1 (Parental)	HEK293/ABCC10 (Resistant)
Paclitaxel	0.073 ± 0.004 (1.00)	0.957 ± 0.044 (13.11)
+Umbralisib 1 μmol/L	0.067 ± 0.009 (0.92)	0.923 ± 0.034 (12.64)
+Umbralisib 3 μmol/L	0.062 ± 0.003 (0.85)	0.939 ± 0.042 (12.86)
+Umbralisib 10 μmol/L	0.066 ± 0.013 (0.90)	0.944 ± 0.038 (12.93)
+Cepharanthine 10 μmol/L	0.075 ± 0.009 (1.03)	0.103 ± 0.083 (1.41)
Cisplatin	1.117 ± 0.059 (1.00)	1.080 ± 0.045 (0.97)
+Umbralisib 1 μmol/L	1.119 ± 0.115 (1.00)	1.124 ± 0.024 (1.01)
+Umbralisib 3 μmol/L	1.014 ± 0.009 (0.91)	1.104 ± 0.058 (0.99)
+Umbralisib 10 μmol/L	0.948 ± 0.037 (0.85)	1.061 ± 0.055 (0.95)
+Cepharanthine 10 μmol/L	0.993 ± 0.027 (0.89)	1.045 ± 0.039 (0.94)

Resistance fold was calculated by dividing the IC_50_ values of substrates in the presence or absence of inhibitor by the IC_50_ of HEK293/pcDNA3.1 cells without inhibitor.

Data are presented as mean ± SD from three independent biological experiments.

### 3.3 Localization and expression of ABCB1 were not affected by umbralisib

**Figure 5:**
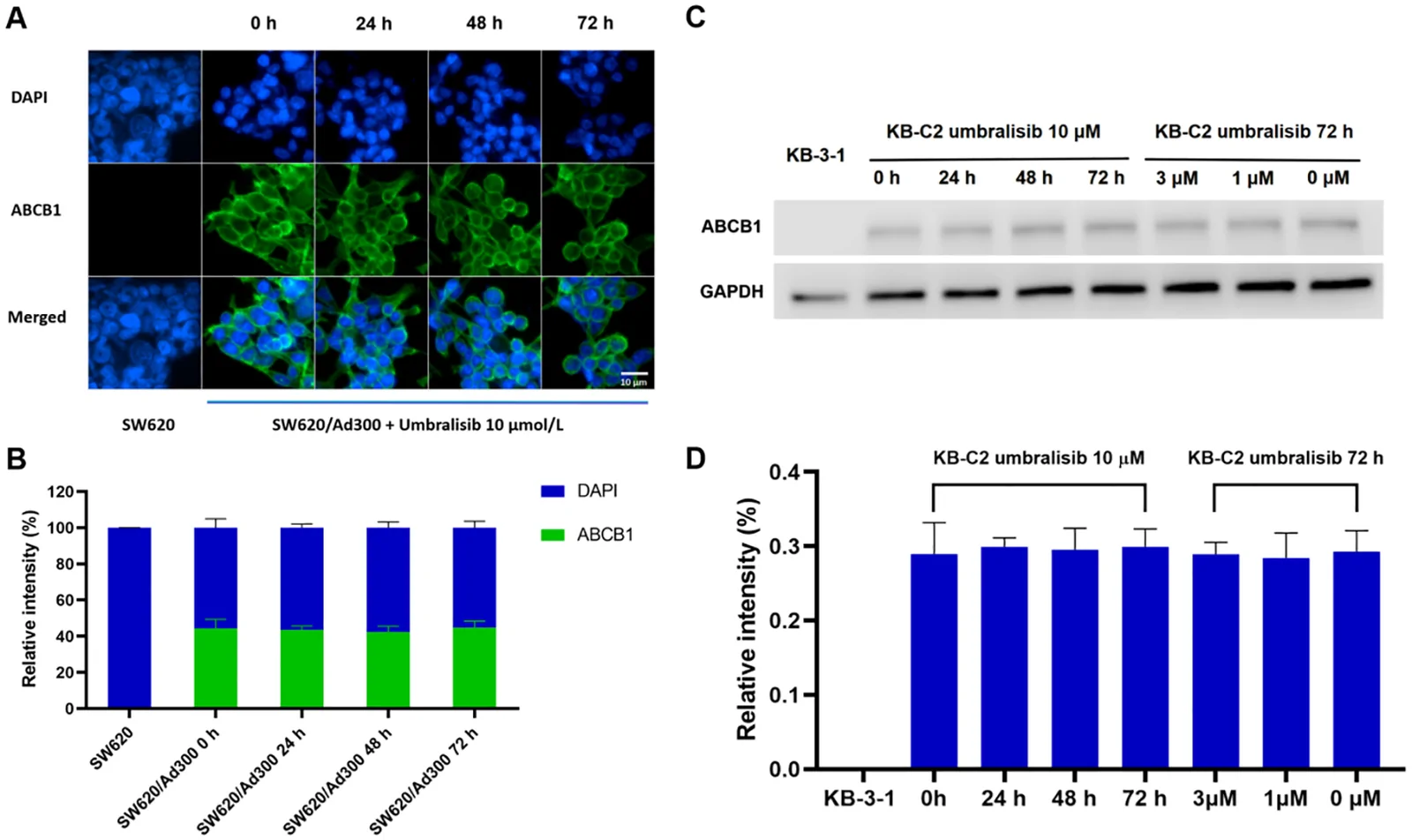
Cellular localization and expression of ABCB1 remained unchanged after umbralisib treatment. **(A)** Immunofluorescence analysis of ABCB1 staining in SW620 and SW620/Ad300 cells treated with 10 μmol/L umbralisib for the indicated times (0, 24, 48, and 72 h). **(B)** Quantification of ABCB1 and DAPI fluorescence intensities corresponding to panel **(A) (C)** Western blot analysis of ABCB1 protein levels in KB-3–1 and KB-C2 cells following umbralisib treatment. KB-C2 cells were exposed either to 10 μmol/L umbralisib for 0–72 h or to increasing concentrations of umbralisib (0, 1, 3, and 10 μmol/L) for 72 h. **(D)** Quantitative analysis of ABCB1 protein levels shown in panel **(C)** Error bars represent SD. Green fluorescence denotes ABCB1, whereas blue fluorescence denotes nuclei. GAPDH served as the internal loading control. The results are representative of three independent experiments. Error bars represent SD.

Previous studies have suggested that reversal of ABCB1-mediated MDR may involve altered cellular localization, reduced expression, or direct inhibition of transporter function (17). To determine whether umbralisib affects ABCB1 localization, an immunofluorescence assay was performed in SW620/Ad300 cells. ABCB1 remained predominantly associated with the cell membrane after treatment with 10 μmol/L umbralisib for 24, 48, or 72 h, and no obvious redistribution was observed. (**Figures 5A, B**). To further examine whether umbralisib altered ABCB1 expression, western blot analysis was performed in KB-C2 cells (**Figure 5C**). The quantification analysis showed that treatment with 10 μmol/L umbralisib for 24, 48, or 72 h did not significantly change the protein level of ABCB1 (**Figure 5D**). Likewise, after 72 h exposure, 1, 3, or 10 μmol/L umbralisib also did not affect ABCB1 expression (**Figure 5D**). Together, these findings suggest that umbralisib did not produce an obvious change in ABCB1 distribution or expression.

### 3.4 Umbralisib increased the intracellular accumulation of [^3^H]-paclitaxel and inhibited its efflux in ABCB1-overexpressing cells

**Figure 6:**
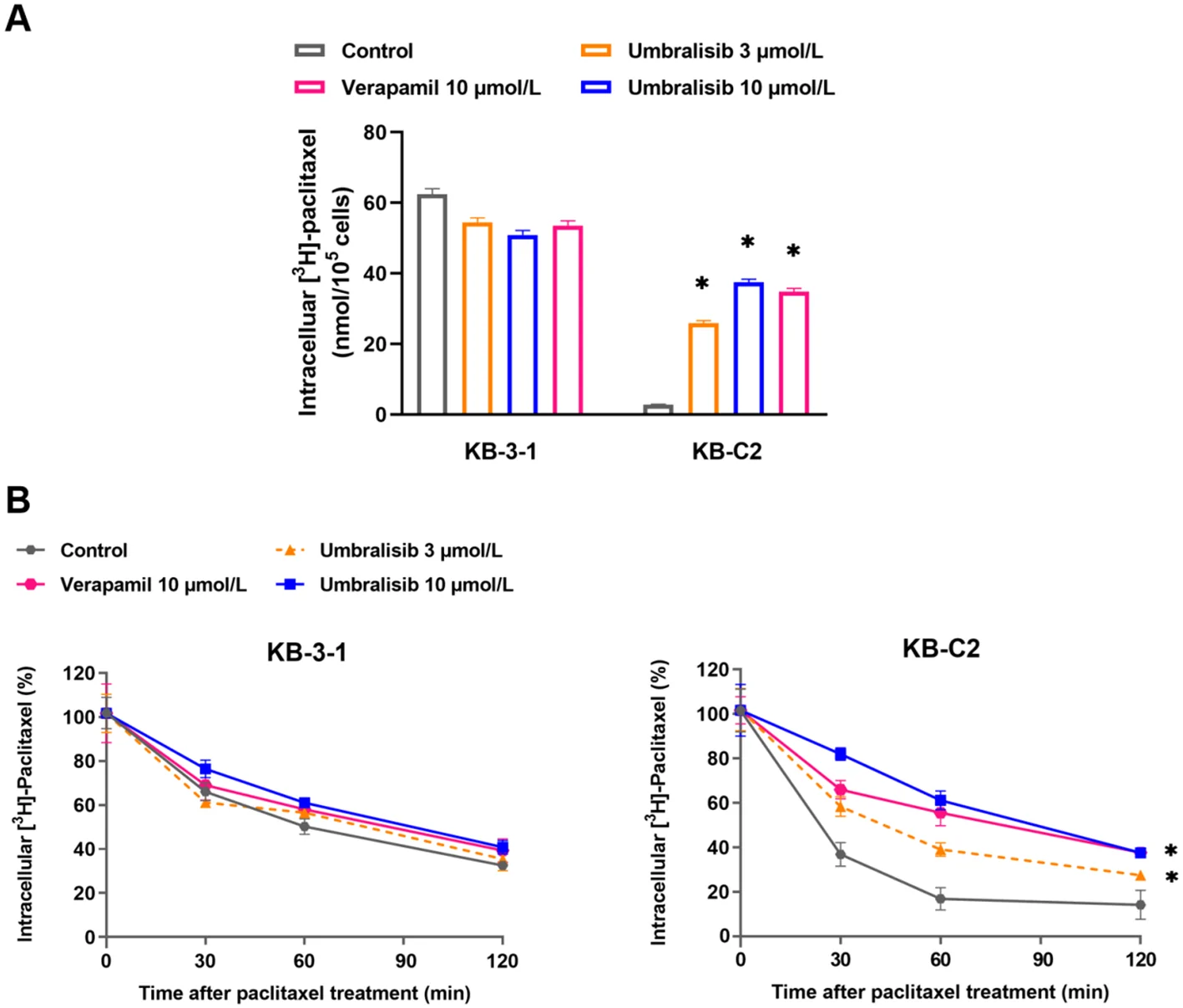
Umbralisib enhanced intracellular retention of [^3^H]-paclitaxel in ABCB1-overexpressing cells by suppressing drug efflux. **(A)** Intracellular accumulation of [³H]-paclitaxel in KB-3–1 and KB-C2 cells after umbralisib treatment. **(B)** Effect of umbralisib on [^3^H]-paclitaxel efflux from KB-3–1 and KB-C2 cells over time. Verapamil (10 μmol/L) was used as the positive control. The results are representative of three independent experiments. Error bars represent SD. *P < 0.05 compared with the control group.

Because the preceding results showed that umbralisib did not significantly change ABCB1 expression or its subcellular distribution, we next examined whether its reversal effect was associated with functional inhibition of transporter activity. Accordingly, [^3^H]-paclitaxel accumulation and efflux assays were carried out. As presented in **Figure 6A**, exposure to umbralisib led to a significant elevation in intracellular [^3^H]-paclitaxel levels in KB-C2 cells, whereas no comparable effect was detected in parental cells. Verapamil, included as the positive control, produced an expected increase in intracellular drug accumulation. These findings indicate that umbralisib promotes retention of ABCB1 substrate drugs in ABCB1-overexpressing cells.

An efflux assay was performed to examine whether the increased intracellular accumulation of paclitaxel was due to reduced ABCB1 transport activity. Umbralisib did not produce a significant effect on [^3^H]-paclitaxel efflux in parental KB-3–1 cells (**Figure 6B**). However, in ABCB1-overexpressing KB-C2 cells, umbralisib significantly delayed [^3^H]-paclitaxel efflux, leading to sustained intracellular retention over time (**Figure 6C**). These data further support that umbralisib reverses ABCB1-mediated MDR by functionally inhibiting ABCB1 efflux.

### 3.5 Molecular docking predicted binding of umbralisib within the ABCB1 drug-binding pocket, and umbralisib modulated ABCB1 ATPase activity

**Figure 7:**
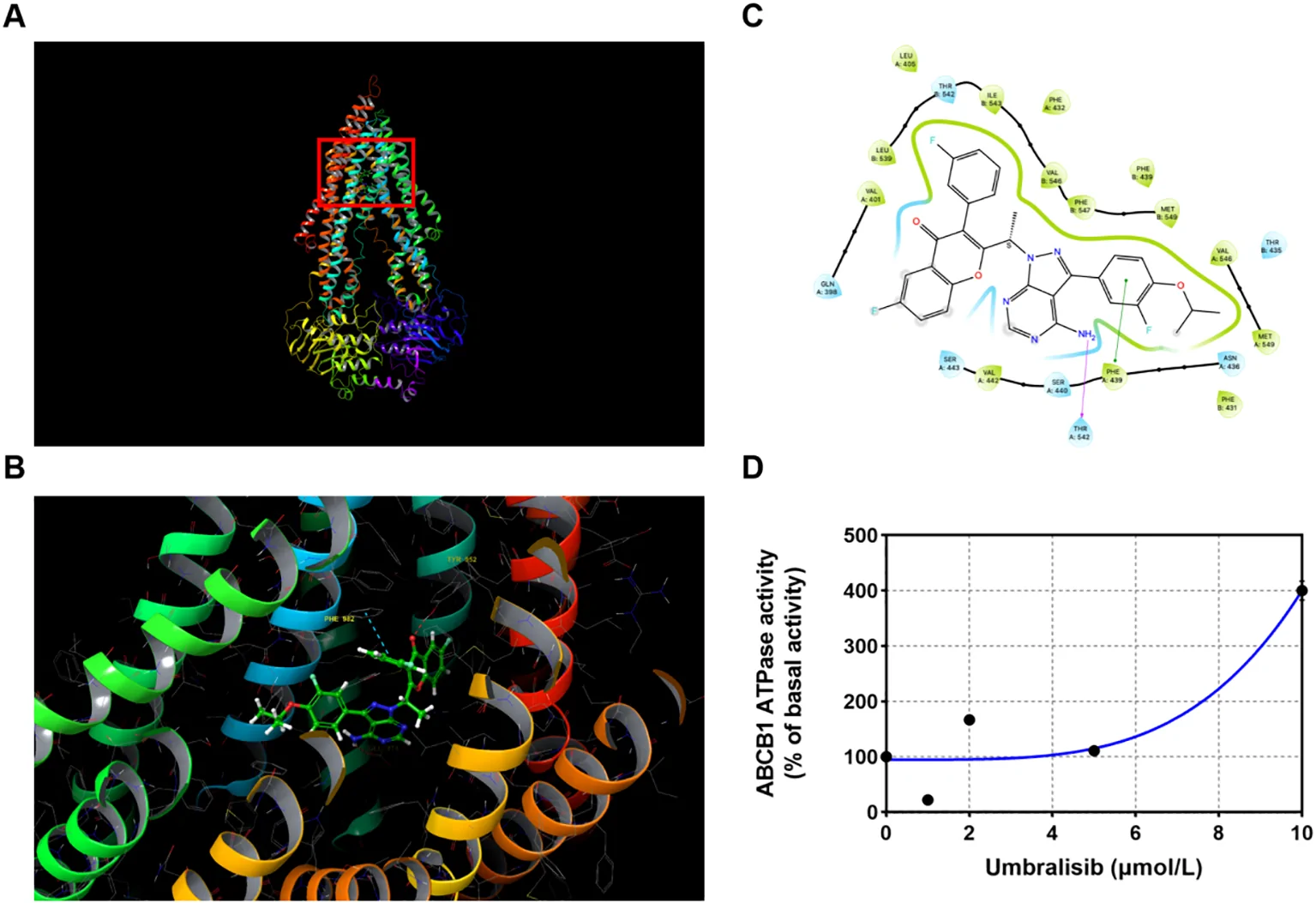
Molecular docking and ATPase analysis of the interaction between umbralisib and ABCB1. **(A)** Predicted binding pose of umbralisib within the drug-binding cavity of human ABCB1. **(B)** Expanded 3D ligand-protein interaction view of umbralisib with ABCB1. **(C)** Corresponding 2D interaction diagram of umbralisib within the ABCB1 binding pocket. **(D)** Stimulatory effect of umbralisib on ABCB1 ATPase activity. Error bars represent SD. The results are representative of three independent experiments. Error bars represent SD. *P < 0.05 compared with the control group.

To further explore the interaction between umbralisib and ABCB1, molecular docking analysis was performed. As shown in **Figures 7A–C**, molecular docking predicted that umbralisib binds within the transmembrane drug-binding cavity of ABCB1, with a favorable docking score of −9.64 kcal/mol (overall docking model in **Figure 7A**; enlarged binding pose in **Figure 7B**). The 2D ligand–receptor interaction diagram in **Figure 7C** indicated that umbralisib formed a hydrogen bond with Thr542 and was surrounded by multiple hydrophobic residues, including Val401, Leu405, Leu539, Ile543, Val546, Met549, Phe432, Phe439, Phe547, Val442, and Phe431. Nearby polar residues, including Gln398, Ser440, Ser443, Asn436, and Thr435, also contributed to the binding site. These docking results supported a predicted binding pose of umbralisib within the ABCB1 drug-binding pocket.

Because the transport function of ABCB1 is driven by ATP hydrolysis, we next evaluated whether umbralisib could affect the ATPase activity of ABCB1. As shown in **Figure 7D**, umbralisib stimulated ABCB1 ATPase activity in a concentration-dependent manner. The concentration-dependent change in vanadate-sensitive ATPase activity was consistent with an ABCB1-associated effect of umbralisib. Although the use of crude membrane vesicles does not establish direct physical binding, reference controls and complementary accumulation and efflux assays support the functional association of umbralisib with ABCB1. Together, these findings indicate that umbralisib attenuates ABCB1-mediated drug efflux.

### 3.6 Umbralisib enhanced paclitaxel-induced apoptosis and restored chemosensitivity in the 3D spheroid model

**Figure 8:**
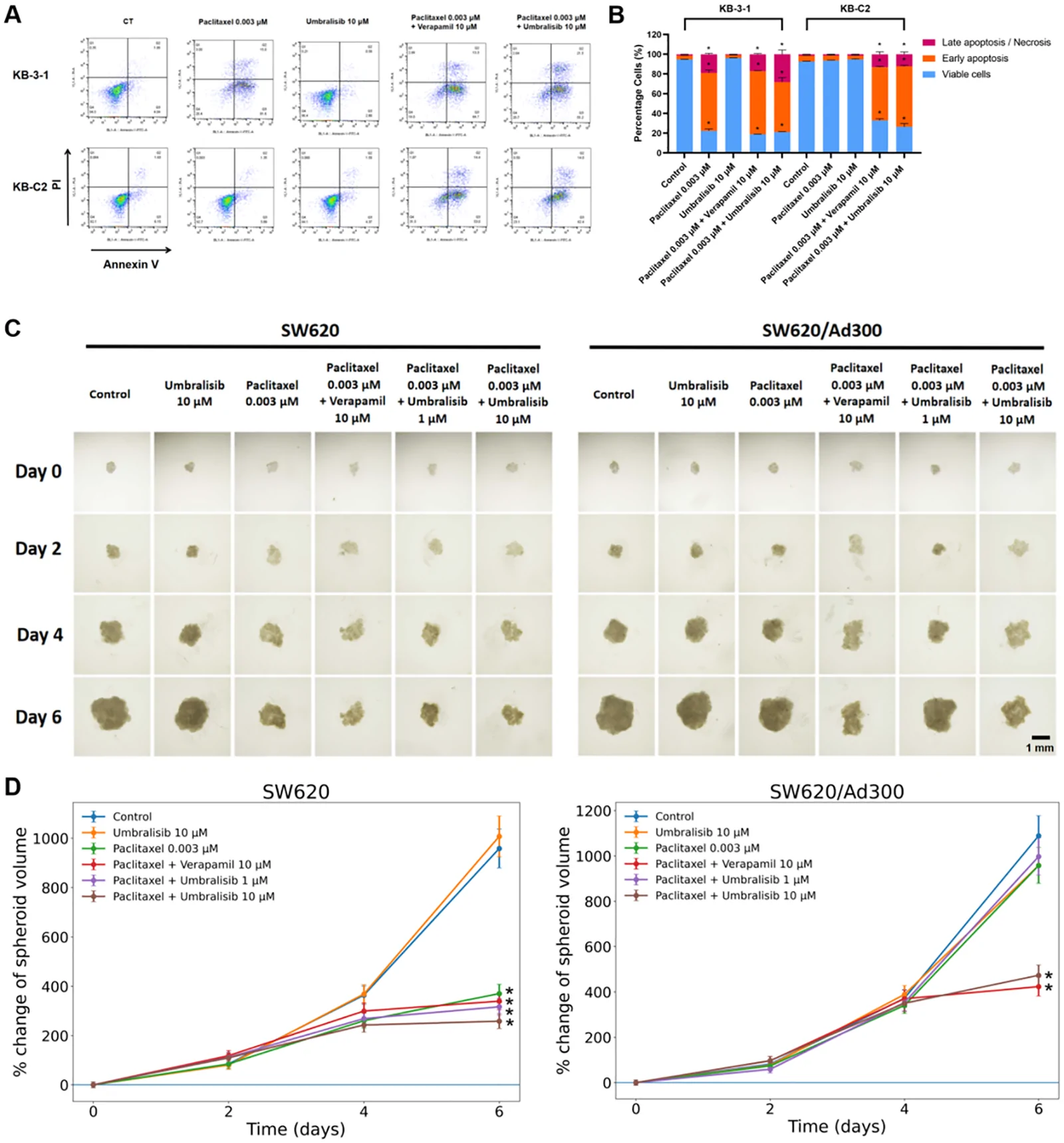
Umbralisib reinforced paclitaxel-induced apoptosis and restored paclitaxel sensitivity in ABCB1-overexpressing cells in 3D systems. **(A)** Representative Annexin V/PI flow cytometry results from KB-3–1 and KB-C2 cells following the indicated treatments. **(B)** Quantification of viable, early apoptotic, and late apoptotic/necrotic fractions, indicating that umbralisib potentiated the pro-apoptotic effect of paclitaxel in KB-C2 cells. **(C)** Representative images of SW620 and SW620/Ad300 spheroids treated for 6 days as indicated. Scale bar = 1 mm. **(D)** Quantitative analysis of spheroid expansion, demonstrating concentration-dependent restoration of paclitaxel sensitivity by umbralisib in SW620/Ad300 spheroids. Data are expressed as mean ± SD obtained from three independent experiments. *P < 0.05 compared with the control group.

To further evaluate whether umbralisib could enhance chemosensitization in ABCB1-overexpressing cells, apoptotic cell death was analyzed in parental KB-3–1 cells and ABCB1-overexpressing KB-C2 cells using Annexin V/PI staining and flow cytometry. Paclitaxel alone induced substantial apoptosis in KB-3–1 cells, whereas the addition of umbralisib did not markedly enhance apoptosis. In contrast, KB-C2 cells showed a limited apoptotic response to paclitaxel alone, which was increased by co-treatment with umbralisib (**Figure 8A**). Quantitative analysis further confirmed that umbralisib significantly increased paclitaxel-induced apoptosis in KB-C2 cells (**Figure 8B**).

The chemosensitizing effect of umbralisib was further examined in a 3D spheroid model using SW620 and SW620/Ad300 cells. SW620 spheroids were sensitive to paclitaxel alone, whereas SW620/Ad300 spheroids showed observable resistance; notably, co-treatment with umbralisib restored paclitaxel responsiveness in SW620/Ad300 spheroids in a concentration-dependent manner (**Figure 8C**). Spheroid volume quantification confirmed this effect, showing that paclitaxel with 10 μmol/L umbralisib produced growth inhibition in SW620/Ad300 spheroids, which was comparable to the positive control group (**Figure 8D**).

## 4 Discussion

Cancer remains a major global health burden, and drug resistance continues to cause treatment failure despite advances in surgery, chemotherapy, and immunotherapy (18). Some research has indicated that overexpression of ABC transporters, especially ABCB1, on the cancer cell membrane contributes to MDR (19, 20). The PI3K signaling pathway plays a critical role in cancer development and progression, and its abnormal activation promotes tumor cell proliferation, survival, metabolic reprogramming, and metastasis (21–23). Previous studies have reported that PI3K inhibitors can reverse ABCB1-overexpression-mediated MDR, supporting their use as chemosensitizers when used together with anticancer drugs (24–26). In addition, dual inhibitors targeting PI3K-related pathways have also been reported to reverse ABCB1-mediated MDR (27, 28). Similarly, CK1ϵ is an oncogenic kinase involved in tumor cell survival, proliferation, and metastatic progression in multiple cancer types, and its inhibitors have also been reported to modulate MDR (29, 30). Therefore, dual PI3K/CK1 inhibitors may have potential in MDR-related cancer research.

Umbralisib is pharmacologically characterized as a dual PI3Kδ/CK1ϵ inhibitor. Although it was withdrawn from clinical use because of safety concerns, our findings indicated that umbralisib significantly reversed ABCB1-mediated MDR (31, 32). In the present study, umbralisib exhibited similar cytotoxicity in both parental and ABCB1-overexpressing cells, including both drug-selected and transfected models, indicating that its reversal effect was not attributable to selective toxicity toward the resistant cells.

After treatment with the known ABCB1 substrate drugs paclitaxel, vincristine, and doxorubicin, ABCB1-overexpressing KB-C2 and SW620/Ad300 cells exhibited resistance compared with their respective parental cells. Co-treatment with umbralisib restored drug sensitivity in ABCB1-overexpressing cells in a concentration-dependent manner. The same phenomenon was also observed in ABCB1-transfected HEK293 cells, further showing that the reversal effect of umbralisib was not limited to drug-selected resistant cells. Validation in the HEK293 transfected model also helps exclude adaptive alterations associated with long-term drug selection and supports that the reversal effect is more directly associated with ABCB1 inhibition by umbralisib.

In contrast, umbralisib did not produce a significant reversal effect in the ABCC1-, ABCG2-, or ABCC10-overexpressing models under the tested conditions. However, because transporter-specific functional assays were not performed for these proteins, these findings do not exclude potential interactions with ABCC1, ABCG2, or ABCC10. The lack of reversal in ABCG2-mediated MDR may be related to the relatively larger molecular structure of umbralisib, which may be less compatible with the narrower substrate-binding cavity of ABCG2 (33).

Reversal agents may overcome MDR by inhibiting transporter function, reducing transporter expression, or altering transporter localization. In our study, Western blot analysis showed that umbralisib did not significantly alter ABCB1 protein levels, indicating that its reversal effect was not mediated by ABCB1 downregulation. Immunofluorescence microscopy further showed that ABCB1 mainly localized on the cell membrane after umbralisib treatment, with no obvious change in cellular distribution. These results indicate that umbralisib does not reverse MDR by reducing ABCB1 expression or altering its membrane localization.

Because ABCB1-mediated MDR is mainly caused by drug efflux, increased intracellular accumulation of ABCB1 substrates and reduced efflux provide direct evidence of transporter inhibition. In our accumulation assays, ABCB1-overexpressing cells showed less intracellular [^3^H]-paclitaxel levels than parental cells, indicating active paclitaxel efflux. Umbralisib increased [^3^H]-paclitaxel accumulation in ABCB1-overexpressing cells, whereas no comparable change was observed in parental cells, supporting inhibition of ABCB1 transport function. In efflux assays, ABCB1-overexpressing KB-C2 cells showed a rapid decrease in intracellular [^3^H]-paclitaxel, whereas umbralisib markedly delayed this process. These findings showed that umbralisib attenuated ABCB1-mediated efflux and thereby increased intracellular retention of substrate drugs.

ATP hydrolysis provides the energy required for ABCB1 transport, and many ABCB1 substrates and modulators alter transporter-associated ATPase activity (34). In the present study, umbralisib produced a concentration-dependent increase in ABCB1 ATPase activity, suggesting a potential interaction with the transporter. ATPase stimulation is commonly interpreted as a sign of the drug-binding cavity, because many transported substrates increase ATP hydrolysis after binding to the transmembrane domain (35). Our ATPase results are also similar to those reported for the PI3K inhibitor BAY-1082439, suggesting that both compounds may engage the drug-binding cavity in a substrate-like manner. However, unlike umbralisib, BAY-1082439 was also reported to reduce ABCB1 expression (24).

The docking results provided computational support for a potential interaction between umbralisib and ABCB1. Computational docking is widely used to examine ligand binding within the transmembrane drug-binding pocket of ABC transporters and to predict residue-level interactions associated with functional effects (36). In our study, umbralisib was positioned within the transmembrane drug-binding cavity of ABCB1, supporting a predicted binding model that may explain the attenuation of efflux activity observed in our experiments. The docking score of -9.64 kcal/mol indicated a favorable predicted binding pose between umbralisib and the transmembrane drug-binding region of ABCB1. The interaction map showed hydrogen bonding together with extensive hydrophobic contacts and π–π interactions, which is consistent with the physicochemical properties of the ABCB1 drug-binding pocket. Together, these structural observations support the accumulation and efflux findings and further suggest that umbralisib may occupy an overlapping region within the drug-binding cavity of ABCB1, thereby interfering with substrate transport.

By inhibiting ABCB1 function, intracellular exposure to substrate drugs is increased, thereby restoring downstream cytotoxic effects such as apoptosis. In our apoptosis studies, treatment with the substrate drug alone produced no significant apoptotic response in ABCB1-overexpressing KB-C2 cells, whereas co-treatment with umbralisib increased the apoptotic population, consistent with restored drug efficacy after efflux inhibition. The increase in apoptotic cells further suggests that umbralisib restored cell death signaling that had been suppressed by ABCB1-mediated drug efflux.

Because 3D tumor spheroids better mimic the *in vivo* tumor environment, they are increasingly used to determine whether reversal effects persist in a more physiologically relevant model (37). In our 3D spheroid model, umbralisib enhanced the growth-inhibitory effect of the substrate drug in SW620/Ad300 resistant spheroids. This result showed that the reversal effect was maintained in a more physiologically relevant model and further confirmed chemosensitization in the 3D spheroid system. Despite the mass transport limitations and structural complexity of 3D tumor spheroids, umbralisib retained chemosensitizing activity in this model.

Previous studies have shown that several PI3K inhibitors can reverse MDR in different cancer models. It has been reported that BAY-1082439 overcomes ABCB1-mediated MDR not only by suppressing PI3K/Akt signaling, but also by directly competing for binding within the transporter cavity (24). In this context, our data extend previous findings on PI3K-related inhibitors by showing that umbralisib modulates ABCB1-mediated drug efflux in several ABCB1-overexpressing models. However, our results were obtained *in vitro*, and clinical translation remains challenging because efforts to inhibit MDR have historically been limited by pharmacokinetic drug interactions and increased toxicity when transporter inhibitors are combined with cytotoxic agents (38, 39). Since umbralisib was withdrawn because of safety concerns, its direct clinical repurposing as an MDR inhibitor would require careful risk-benefit assessment. At this stage, it may be more useful for mechanistic studies, lead optimization, or transporter-drug interaction prediction than for immediate clinical translation. Although the unbound plasma concentration of umbralisib is expected to be lower than its total plasma exposure because of extensive protein binding, the concentration range examined in this study remains informative for defining its concentration-dependent modulation of ABCB1 function. These findings therefore provide pharmacologically relevant mechanistic evidence, while their direct clinical translation requires further evaluation. Moreover, because MDR is driven by multiple mechanisms, inhibition of transporter function alone may not be sufficient to fully reverse resistance. Nevertheless, this study provides mechanistic evidence that umbralisib can modulate ABCB1-mediated drug efflux and may help inform the investigation of other compounds with related structural or pharmacological properties.

In summary, umbralisib reversed ABCB1-mediated MDR in a concentration-dependent manner, primarily by attenuating ABCB1 efflux activity. This conclusion was supported by MTT-based reversal assays, [^3^H]-substrate accumulation and efflux assays, ATPase analysis, and molecular docking simulation, while Western blotting and immunofluorescence analyses revealed no significant change in ABCB1 expression or membrane localization. The increased apoptotic response and the sustained reversal effect in the 3D spheroid model further showed that this inhibition restored chemosensitivity in both 2D and 3D models. Overall, these findings provide a rationale for further evaluation of umbralisib in ABCB1-mediated MDR research.

## 5 Conclusion

In summary, umbralisib reversed ABCB1-mediated multidrug resistance primarily by attenuating ABCB1-dependent drug efflux without altering transporter expression or cellular distribution. This effect increased intracellular drug retention, restored paclitaxel-induced apoptosis, and remained evident in the 3D spheroid model. Although the clinical withdrawal of umbralisib limits its immediate therapeutic application, the present findings identify an additional pharmacological property of this compound and provide mechanistic insight into ABCB1 modulation that may inform the evaluation of related compounds with improved safety profiles.

## Data Availability

The raw data supporting the conclusions of this article will be made available by the authors, without undue reservation.
